# Integrative genomic analyses identify neuroblastoma risk genes involved in neuronal differentiation

**DOI:** 10.1007/s00439-024-02700-2

**Published:** 2024-08-27

**Authors:** Matilde Tirelli, Ferdinando Bonfiglio, Sueva Cantalupo, Annalaura Montella, Marianna Avitabile, Teresa Maiorino, Sharon J. Diskin, Achille Iolascon, Mario Capasso

**Affiliations:** 1 Department of Molecular Medicine and Medical Biotechnology, University of Naples Federico II, 80131 Naples, Italy; 2 CEINGE Biotecnologie Avanzate Franco Salvatore, 80145 Naples, Italy; 3 Division of Oncology and Center for Childhood Cancer Research, Children’s Hospital of Philadelphia, 19104 Philadelphia, USA; 4 Department of Pediatrics, Perelman School of Medicine, University of Pennsylvania, 19104 Philadelphia, USA

## Abstract

Genome-Wide Association Studies (GWAS) have been decisive in elucidating the genetic predisposition of neuroblastoma (NB). The majority of genetic variants identified in GWAS are found in non-coding regions, suggesting that they can be causative of pathogenic dysregulations of gene expression. Nonetheless, pinpointing the potential causal genes within implicated genetic loci remains a major challenge. In this study, we integrated NB GWAS and expression Quantitative Trait Loci (eQTL) data from adrenal gland to identify candidate genes impacting NB susceptibility. We found that *ZMYM1*, *CBL*, *GSKIP* and *WDR81* expression was dysregulated by NB predisposing variants. We further investigated the functional role of the identified genes through computational analysis of RNA sequencing (RNA-seq) data from single-cell and whole-tissue samples of NB, neural crest, and adrenal gland tissues, as well as through in vitro differentiation assays in NB cell cultures. Our results indicate that dysregulation of *ZMYM1*, *CBL*, *GSKIP*, *WDR81* may lead to malignant transformation by affecting early and late stages of normal program of neuronal differentiation. Our findings enhance the understanding of how specific genes contribute to NB pathogenesis by highlighting their influence on neuronal differentiation and emphasizing the impact of genetic risk variants on the regulation of genes involved in critical biological processes.

**Supplementary Information:**

The online version contains supplementary material available at 10.1007/s00439-024-02700-2.

## Introduction

Neuroblastoma (NB) is a pediatric malignancy that emerges from defective differentiation of neural crest-derived cells of the sympathoadrenal lineage of the nervous system (Matthay et al. 2016). Clinical and biological factors play a critical role in stratifying risk and formulating patients’ treatment strategies, taking into account features including age at diagnosis, cancer stage, tumor histopathology, ploidy, and genomic abnormalities (Matthay et al. 2016). In young children, adverse prognostic features have been associated with *MYCN* amplification and arm-level chromosomal alterations, such as deletions in 1p and 11q, unbalanced gains in 17q, and rearrangements in *TERT* (Capasso and Diskin 2010). In contrast, children over the age of six often exhibit unique structural variants, with the most frequent ones being 19p loss and 1q gain (Lasorsa et al. 2020). Primary tumors harbor somatic coding and non-coding point mutations in genes like *ALK* and *ATRX*, as well as in regulatory elements. These mutations are considered cancer drivers and hold the potential to predict patient prognosis (Pugh et al. 2013; Capasso et al. 2020; Lasorsa et al. 2022). In addition, we recently identified the N546K mutation in the *FGFR1* gene, which activates its downstream pathways (Cimmino et al. 2022). Also, gene expression signatures have emerged as valuable tools for predicting clinical outcomes in high-risk patients (Barbieri et al. 2013; Formicola et al. 2016). Nevertheless, despite the strides made in identifying clinically actionable genomic alterations, NB still accounts for 15% of all childhood cancer-related deaths, with less than 40% of affected children surviving beyond 5 years (Park et al. 2017).

Sporadic and familial NB account for approximately 98% and 2% of NB cases. Germline and somatic mutations contribute to the development of both forms of NB (Tonini and Capasso 2020). Recent advances in high-throughput methodologies and genome-wide analyses have significantly expanded our knowledge of NB genetics and helped to decipher, although not completely, the specific contribution of germline variation to disease susceptibility (Tonini and Capasso 2020; Bonfiglio et al. 2023). Specifically, genome-wide association studies (GWAS) identified single nucleotide polymorphisms (SNPs) associated with increased NB risk and contributed to undercover the molecular mechanisms of NB pathogenesis through the discovery of candidate genes which may impact disease risk. Of note, a NB susceptibility locus on chromosome 6p22 harboring a SNP within the long non-coding RNA (lncRNA) CASC15 produces a truncated isoform whose reduced expression correlates with advanced disease (Maris et al. 2008; Russell et al. 2015). In contrast, the loss of NBAT-1, another lncRNA mapping to the same locus, has been shown to hinder the differentiation of neuronal progenitors and linked to a more aggressive phenotype (Pandey et al. 2014). SNPs in *BARD1* were found to exert different effects on the protein function and therefore affect NB pathogenesis (Capasso et al. 2009; Bosse et al. 2012; Cimmino et al. 2018, 2020). Also, SNPs in *LMO1,* causing a decreased expression of the encoded gene, correlated with reduced cell proliferation (Wang et al. 2011), while genetic variants activating *LIN28B* promoted tumorigenesis of aggressive NB (Diskin et al. 2012). The investigation on other susceptibility genes, including *MLF1*, *CPZ*, *NEFL*, *CDKN1B* (Capasso et al. 2014, 2017; McDaniel et al. 2017), also outlined the importance of the functional characterization of SNPs to specify novel biological pathways implicated in NB tumorigenesis.

Although GWASs have been pivotal in mapping chromosomal loci linked to complex traits and in identifying genetic variants involved in susceptibility to NB and other pediatric cancers (Sweet-Cordero and Biegel 2019), they do exhibit certain limitations. Notably, many of these variants maps to non-coding regions of the genome. This complicates the fine-mapping of which specific genes may be affected by expression changes and the physiological consequences of such alterations. Therefore, to fully understand the impact of these variants, it is essential to integrate GWAS findings with additional *-omic* data, such as gene expression from disease-relevant tissues to obtain Quantitative Trait Loci (eQTLs). This approach, also known as Transcription-Wide Association Studies (TWAS), can facilitate the identification of SNPs that most likely play a functional role in gene regulatory networks (Mai et al. 2023).

In line with these observations, we hypothesize that GWAS risk variants could lead to expression changes in genes that are critical for neuronal differentiation, a central process in NB pathogenesis contributing to disease initiation (Zeineldin et al. 2022). Here, we employed a computational approach that integrates NB GWAS and eQTL data from adrenal gland which is the most common site of NB occurrence (Matthay et al. 2016). The adrenal gland was chosen as the disease model for this study due to its origin from neural crest-derived precursors of sympathetic neurons and adrenal chromaffin cells (Jansky et al. 2021).

Through our integrative approach, we aimed to examine the expression of candidate risk genes during the differentiation process, thereby uncovering additional elements within the signaling networks driving NB.

## Material and methods

### GWAS summary statistics

Summary statistics were obtained from the latest NB GWAS, encompassing 2101 NB cases and 4202 controls (McDaniel et al. 2017), deposited in dbGaP (accession number phs000124). We filtered out SNP markers with a minor allele frequency (MAF) below 0.01 to focus on common variants. We excluded SNPs with an imputation quality score (INFO) below 0.7 to ensure high-quality imputed data. After these filtering steps, we retained a total of 7,968,526 high-quality SNPs for our downstream analyses.

### Colocalization analysis

In order to find SNPs in NB risk regions that may influence the expression of their target gene (eQTL), we carried out colocalization analysis with Sherlock (He et al. 2013). The Sherlock integrative framework investigates possible causal links between disease and gene expression impacted by eQTLs. Using a Bayesian statistical framework, the method integrates summary-based results of eQTLs and SNP association signals from GWAS. For each tested gene, the Sherlock integrative analysis tool provides a score as Logarithm of Bayes Factor (LBF), which estimates the probability of a gene-suicide relationship, and p-value. Sherlock analysis was carried out using the default parameters and setting the disease prevalence at 1/10,000 (Int. J. Cancer: 135, 2249–2261 (2014)) using the NB GWAS summary statistics described above and GTEx v7 eQTLs from adrenal gland computed from 175 samples.

### MetaXcan TWAS analysis

MetaXcan (Barbeira et al. 2018) was used to impute trait-associated differential gene expression using reference data with available gene expression and genotyping such as GTEx. MetaXcan is a gene-level association approach that tests the mediating effects of gene expression levels on the phenotype. In order to impute differential expression from the adrenal gland tissue (n = 175), we used Multivariate Adaptive Shrinkage in R (MASHR) prediction models available for the GTEx v8 on www.predictdb.org in the form of SQLite files. These prediction models, informed by posterior causal probability from fine-mapping and borrowing information across tissues, have shown better performance in terms of number of significant associations compared to the standard models and have been shown to improve the reliability of causal gene identification (He et al. 2020). Before running MetaXcan analysis, the NB GWAS summary statistics were harmonized, imputed and lifted over to build 38 to match the reference model according to the author’s guidelines (instructions and codes are available at https://github.com/hakyimlab/MetaXcan).

### Gene expression analysis of bulk and single-cell RNA-seq

Bulk gene expression data were downloaded from the R2: Genomics Analysis and Visualization Platform. Specifically the following datasets were downloaded: Normal adrenal gland (GSE3526, GSE7307, GSE8514, n = 13); Normal tissues (GSE3526, n = 353) (Roth et al. 2006); Normal neural crest (GSE14340, n = 5) (de Pontual et al. 2009); Neural precursor cells (GSE7178, n = 9); Tumor neuroblastoma (GSE14880, n = 34); Tumor neuroblastoma (GSE16237, n = 51); Tumor neuroblastoma (GSE13136, n = 30) (Lastowska et al. 2007); Tumor neuroblastoma (GSE16476, n = 88) (Molenaar et al. 2012); Xenograft neuroblastoma (GSE90121, n = 16) (Seong et al. 2017). Data were grouped into three classes: “Differentiated tissues”, including Normal adrenal gland and Normal tissues datasets; “Undifferentiated tissues”, including Normal neural crests and Neural precursor cells; “Tumor”, including Tumor neuroblastoma and Xenograft neuroblastoma datasets. Differences among mean of each group were evaluated through Wilcoxon-Mann–Whitney test.

The same platform was used to retrieve data from all-trans retinoic acid (RA) differentiating treatment on SH-SY5Y (GSE9169, n = 86) (Nishida et al. 2008). Differences between gene expression and treatment time-points were tested using linear regression.

Normalized gene expression data of *ZMYM1*, *CBL*, *GSKIP* and *WDR81* from 496 samples profiled by RNAseq (GEO ID: GSE62564), have been downloaded by R2: Genomics Analysis and Visualization Platform. Mann–Whitney U-Test was used to compare differences in gene expression levels between groups.

Single-cell gene expression data of a reference atlas of the human fetal adrenal medulla were obtained from Jansky et al. (2021). Precomputed labels were used to identify cell subsets of interest—Schwann cell precursors (SCPs), late chromaffin cells, late neuroblasts—and transcriptional changes among them assessed with the MAST method (Finak et al. 2015) specifically suited for the bimodal distribution of single-cell RNA-seq data.

After multiple testing correction using the Benjamini–Hochberg method, we set the false discovery rate (FDR) threshold of 0.05. All statistical analyses were carried out in the R environment and all visualizations were performed using the ggplot2 package.

### Cell culture and differentiating treatments in vitro

The human SH-SY5Y and SK-N-BE(2) cell lines were cultured in Dulbecco’s modified Eagle’s medium (DMEM) and DMEM/Nutrient Mixture F-12 (F-12), respectively. Medium was supplemented with 10% heat-inactivated fetal bovine serum (FBS) (Sigma), 1 mM l-glutamine, penicillin (100 U/ml) and streptomycin (100 µg/ml) (Invitrogen), and cultured at 37 °C, under 5% CO_2_ in a humidified atmosphere.

To induce differentiation, all-trans retinoic acid (RA) (Sigma) and brain-derived neurotrophic factor (BDNF) (Merck) were employed. The differentiation protocol used is described by Shipley et al. (2016). Briefly, cells were seeded in their culture medium to reach confluence level of about 40–50%; on day 1, the medium was exchanged to serum-deprived medium with RA addition dissolved in DMSO to a final concentration of 10 µM. Medium containing RA was changed every 2 days. RA-differentiating cultured cells were carried out until day 5 from seeding and cells were harvested for protein and RNA extraction at each time-point (1d, 2d, 3d, 5d).

For BDNF-induced differentiation, a two-step protocol was used as described by Encinas et al. (2000). Briefly, cells were cultured following the RA-differentiating protocol until day 5 from seeding; then, medium containing RA was removed and changed in serum-deprived medium with BDNF addition at a final concentration of 50 ng/ml. Cells were harvested at day 3 following BDNF addition for protein and RNA isolation.

### Western blotting

Cell pellets were lysed in RIPA buffer (50 mM Tris–HCl, pH 7.5, 150 mM NaCl, 1% Triton X-100, 10% glycerol), complemented with protease inhibitors (ThermoScientific). Protein concentrations were determined by using Bradford assay (Bio-Rad). 30–40 μg of whole-cell lysate proteins were separated on an SDS-PAGE gel and blotted onto an Immobilon-P membrane (Merck). Blocking was performed through 5% non-fat dried milk (EuroClone) in Tris-buffered saline with 0.1% Tween (TBS-T) for 1 h. Then, membranes were incubated with primary antibodies at 4 °C overnight. Incubation with secondary antibodies horseradish-peroxidase-conjugated (anti-rabbit and anti-mouse) was performed for 1 h and the positive bands were visualized using the ECL kit SuperSignalTM West Pico PLUS Chemiluminescent Substrate (Thermo Scientific). Primary antibodies used are the following: anti-ZMYM1, PA5-21,089 (Invitrogen); anti-CBL, 2179S (Cell Signaling); anti-WDR81, 24,874–1-AP; anti-GSKIP(C14orf129), MA5-24550 (Thermo Scientific); anti-NEFL, HPA014850 (Sigma); anti-NeuN, 1294S (Cell Signaling); anti-β-actin, A5441 (Sigma).

### Real-Time PCR

Total RNA extraction was performed using TRIzol LS Reagent (Invitrogen), then cDNA retrotranscription using the High-Capacity cDNA Reverse Transcription Script (Applied Biosistem) was performed according to the manufacturer’s protocol. Specific primers for the investigated genes were designed by PRIMEREXPRESS software (Applied Biosystems) and are listed below:

ZMYM1

For: 5ʹ-AGACACCGATGTTGCCTTGCC-3ʹ; Rev: 5ʹ-GTTCCGTACTACTACCGCTTGGCT.

CBL

For: 5ʹ-AGGGAGACACATTTCGGATTAC-3ʹ; Rev: 5ʹAAGCTCTTCCAAGGGACTATTG-3ʹ

WDR81

For: 5ʹCGCCTGCTGACATCTTGTTA-3ʹ; Rev: 5ʹ-GGGCAGGTACTGGTAGGTGA-3ʹ

GSKIP

For: 5ʹ-ACATGAGGCTCGAAGCTGAA-3ʹ; Rev 5ʹ-TTCCACATTGATATAGGCCACA-3ʹ

NEFL

For 5ʹ- GCATAACCAGTGGCTACTCCC-3ʹ; Rev 5ʹ- TCCTTGGCAGCTTCTTCCTCTTC-3ʹ

SYNAPTOPHYSIN

For 5′-CTTTCTGGTACAGCCGTGAG-3′; Rev 5′-ACAGGGTCCCTCAGTTCCTT-3′

TAU

For: 5ʹ-CTGAGGAACCGGGCTCTGA-3ʹ; Rev: 5ʹ-CCTCATCCACTAAGGGTGCTGT-3ʹ

β-Actin

For: 5′-CGTGCTGCTGACCGAGG-3′;

Rev: 5′-GAAGGTCTCAAACATGATCTGGGT-3′)

SYBR Green PCR Master Mix (AppliedBiosystems) was used for Real-Time PCR and the experiments were carried out in the 7900HT Fast Real-Time PCR System (Applied Biosystems). Relative gene expression was obtained applying the 2 − ΔCT method, where the ΔCT was calculated using the differences in the mean CT between the selected genes and the internal control (β-actin). Each experiment was carried out in triplicate.

### Densitometric analysis

Densitometry of immunoblots was performed with ImageJ software utilizing established methods. Blots were imported to ImageJ and utilizing the ‘analyze gel’ feature, the areas under the curves generated from the bands were obtained for further analysis.

## Results

### Colocalization analysis identified NB risk genes

**Fig. 1:**
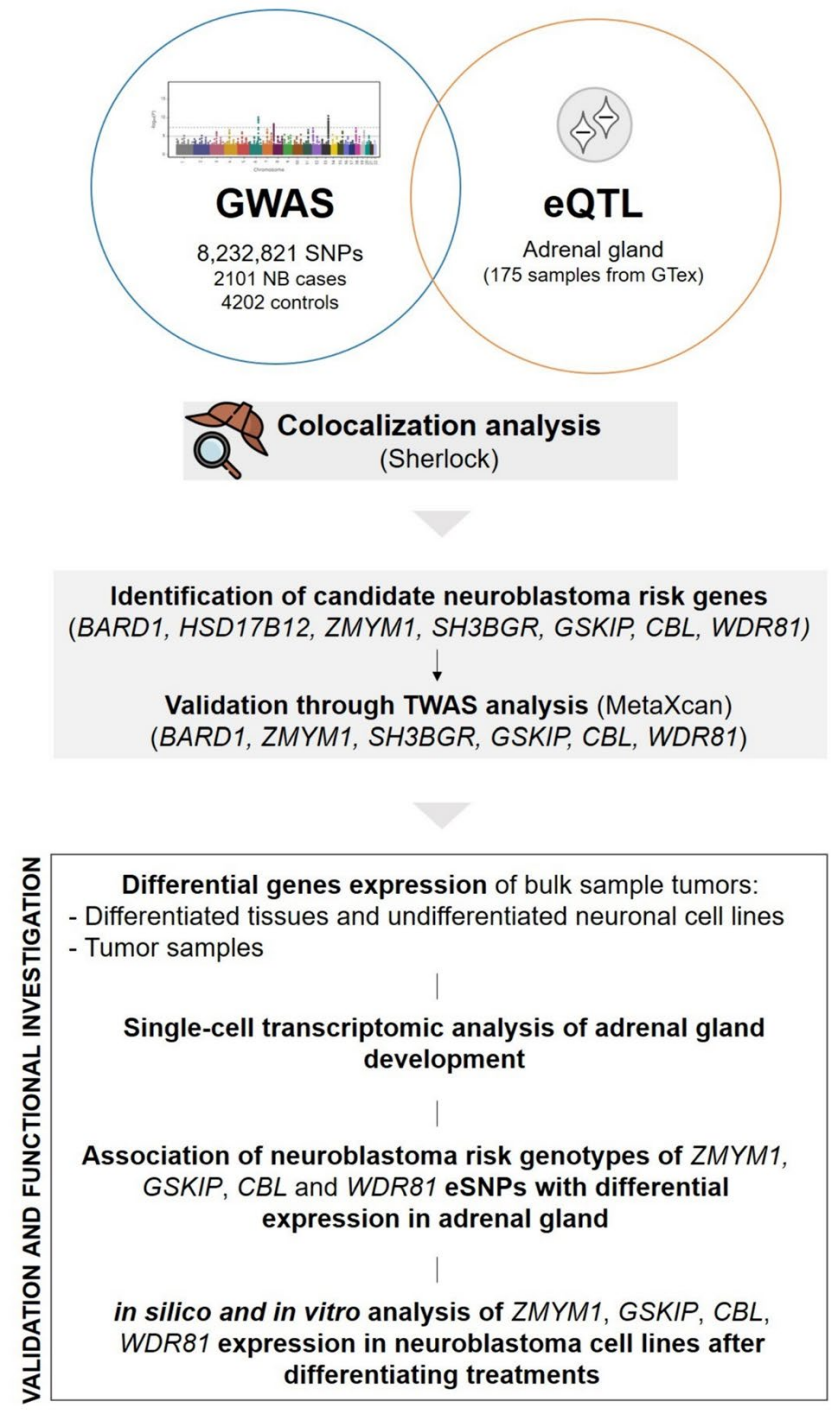
Schematic workflow of NB risk genes identification. Candidate genes identified by colocalization analysis (Sherlock) by matching GWAS data from 8,232,821 SNPs (2101 NB patients and 4202 controls) and eQTL data in adrenal gland (175 samples from GTEx) were further validated through MetaXcan analysis. Subsequent investigation on the identified genes suggested that *ZMYM*, *GSKIP*, *CBL* and *WDR81* may represent promising NB risk genes. eSNPs: expression SNPs

We exploited summary statistics from a GWAS study involving 2101 NB cases and 4202 healthy controls (McDaniel et al. 2017) (Fig. 1).

To assess the impact of risk variants on gene expression, we used a Bayesian method (Sherlock), to integrate NB risk SNPs with eQTL data from 175 adrenal gland samples from GTEx consortium (Fig. 1**)**. This approach prioritized functionally relevant risk variants predicted to affect gene expression. We pinpointed the top candidate genes (*BARD1*, *HSD17B12*, *ZMYM1*, *SH3BGR*, *GSKIP*, *CBL*, *WDR81*), showing P-value lower than 0.001, whose expression changes were predicted to affect NB risk. For each gene, we identified a specific SNP (referred to as “expression SNP” or “eSNP”) that exhibited significant associations with both NB risk and the respective target gene expression (P-value < 0.001) (Table 1). The presence of these eSNPs can potentially impact NB risk by regulating changes in the expression of their associated genes.

**Table 1:** Top candidate genes from colocalization analysis (Sherlock, left) and validation through TWAS analysis (MetaXcan, right)

Gene	LBF^a^	P-value^b^	Supporting SNP^c^	P_eQTL_^d^	P_GWAS_^e^	Zscore^f^	P-value^g^
*BARD1*	7.441	2.88E-05	rs13021937	7.61E-08	1.07E-08	–5.426	5.75E-08
*HSD17B12*	5.7133	0.00013	rs1878764	3.30E-06	2.46E-07	–0.048	9.62E-01
*ZMYM1*	5.349	0.0002	rs3863722	5.22E-09	0.00014	3.249	1.16E-03
*SH3BGR*	4.7957	0.00043	rs2205200	1.02E-11	0.00014	3.341	8.35E-04
*GSKIP*	4.5396	0.00059	rs3759601	9.08E-16	0.00025	2.984	2.84E-03
*CBL*	4.2612	0.00081	rs1893034	3.64E-07	0.0007	–2.477	1.32E-02
*WDR81*	4.1294	0.00096	rs12937244	5.44E-06	0.00069	3.381	7.23E-04

^a^LBF: Logarithm of the Bayes Factor for the gene obtained from colocalization analysis (Sherlock). Higher LBF is associated with higher probability that the gene is associated with NB. For a given gene, Sherlock first identifies all eSNPs and then scores each eSNP based on the significance of the association between the eSNP and NB (positive score: the eSNP is significantly associated with NB; negative score: the eSNP is not associated with NB). The sum of the LBFs for each eSNP of each gene was used as the final LBF score of the given gene

^b^P-value from Sherlock statistical inferences

^c^Only the SNP with the highest LBF is listed

^d^P-value from expression quantitative trait analysis

^e^P-value from GWAS

^f^Zscore from TWAS analysis (MetaXcan)

^g^P-value from TWAS analysis (MetaXcan)

### MetaXcan confirmed candidate NB risk genes identified by colocalization analysis

To confirm previous findings, we carried out a TWAS analysis with MetaXcan using eQTLs of adrenal gland tissues included in GTEx v8 (Fig. 1). This strategy provided concordant results for six out of seven previously identified genes relating to NB risk (*BARD1, ZMYM1, SH3BGR, GSKIP, CBL, WDR81*) (Table 1).

The results obtained from the application of two independent integrative methods consistently strengthen the potential involvement of these genes in contributing to NB risk. We excluded *BARD1* from further analyses as it has been extensively studied in our previous research (Cimmino et al. 2017, 2018, 2020). *HSD17B12* was not validated by the MetaXcan analysis. Therefore, we have excluded it from further investigation within this manuscript.

### Expression analysis of candidate NB risk genes showed differences in undifferentiated neuronal cell lines and tumor tissues compared to differentiated tissues

**Fig. 2:**
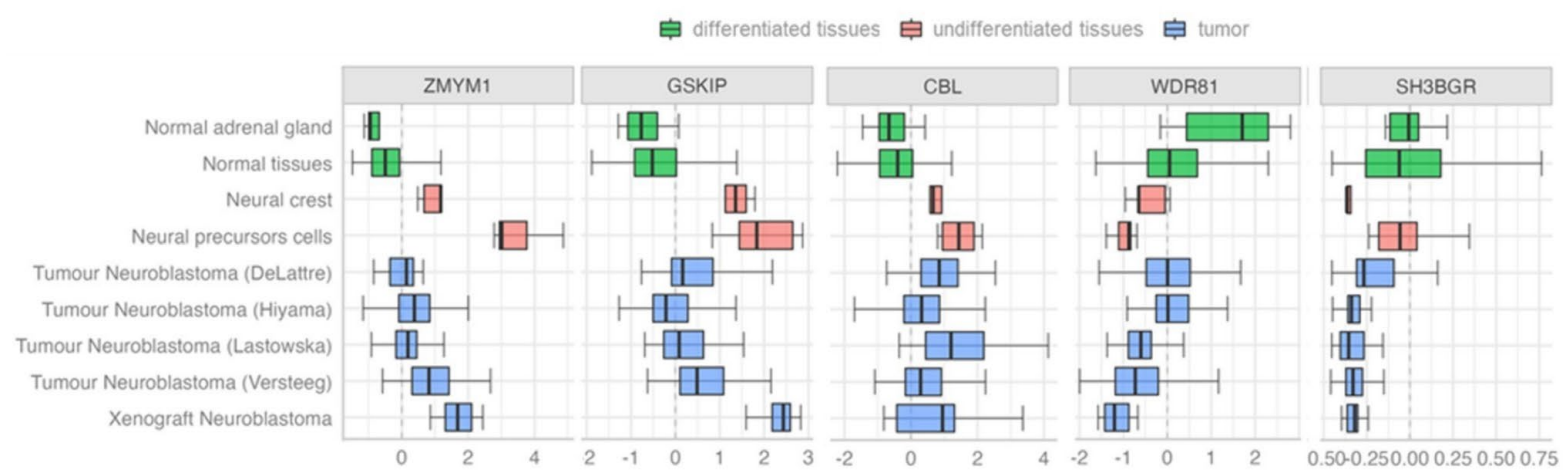
Candidate NB risk genes differential expression between differentiated and undifferentiated tissues. Boxplots show genes expression from public datasets in differentiated tissues (including normal adrenal gland and normal tissues, in green), undifferentiated tissues (including neural crest and neural precursors cells, in pink) and tumor tissues (including primary tumors and xenograft, in blue). P-value and statistics are reported in Supplementary Table 1

NB originates from a defective differentiation of neural crest-derived cells within the sympathoadrenal lineage of the nervous system (Matthay et al. 2016). Starting from this evidence, we evaluated gene expression levels in several NB related-tissues from publicly available datasets **(**Fig. 1). Specifically, we examined the expression of *ZMYM1*, *SH3BGR*, *GSKIP*, *CBL*, and *WDR81* in: (i) differentiated tissues, including adrenal gland and other normal tissues, (ii) undifferentiated tissues, encompassing neural crest cell lines and neural precursor cells, (iii) tumor tissues, involving NB tumors and NB xenografts (Fig. 2 and Supplementary Table 1).

Our analysis showed that *ZMYM1*, *GSKIP,* and *CBL* were highly expressed in neural crest and neural precursor cells, which may resemble undifferentiated status that ultimately give rise to NB. We also found higher expression of these genes in NB tumors and xenografts, when compared with adrenal gland and normal tissues as reported in Fig. 2 (number of samples per group and p-values are reported in Supplementary Table 1). In contrast, *WDR81* showed lower expression in neural crest cells, neural precursor cells, and NB tumors compared to differentiated tissues (Fig. 2 and Supplementary Table 1). *SH3BGR* exhibited only weak associations among the tissue groups and was found to be expressed at very low levels (Fig. 2 and Supplementary Table 1), leading us to omit this gene from subsequent analyses.

**Fig. 3:**
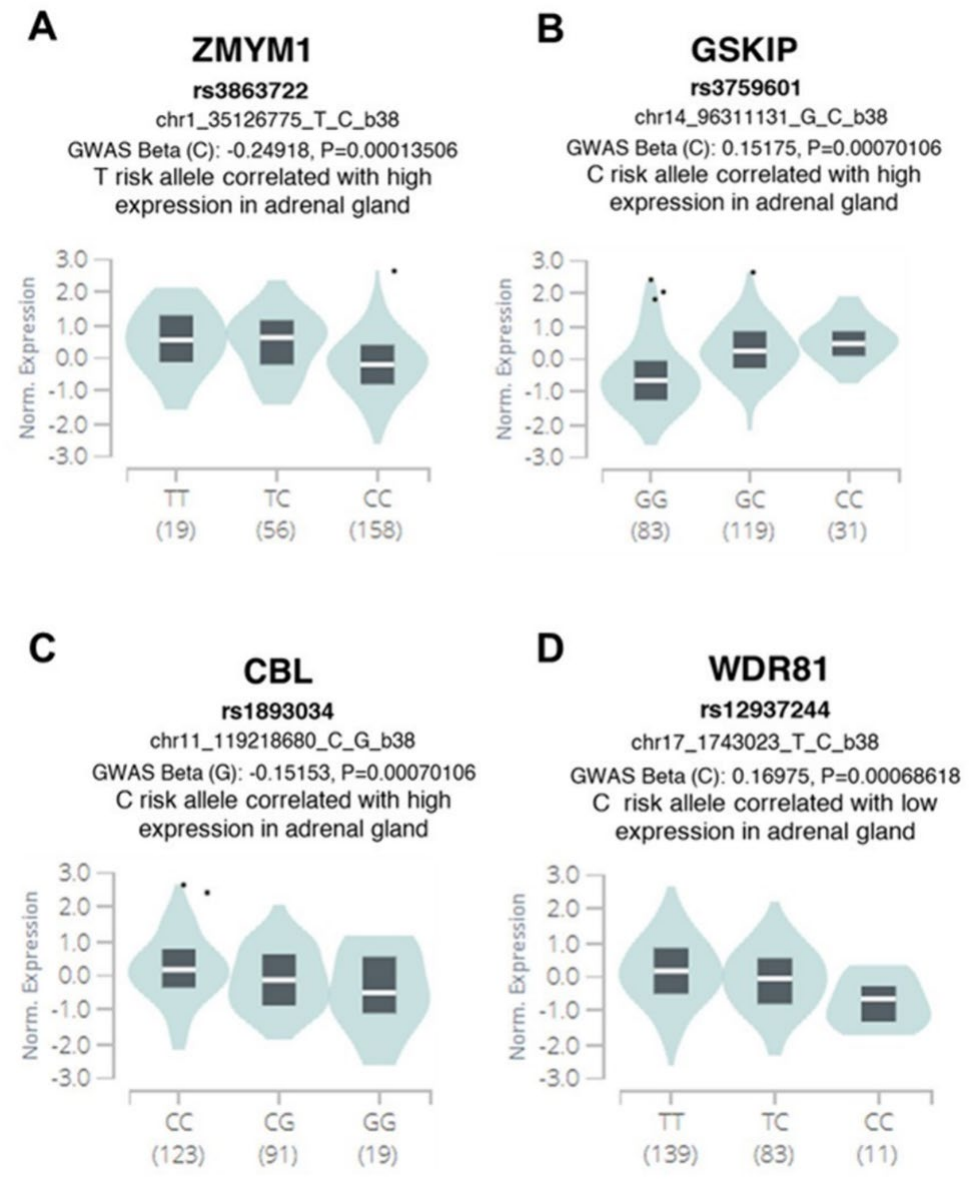
eQTL risk genotypes of the identified candidate genes in association with their expression in adrenal gland tissue**.** Violin plots show the median expressions of *ZMYM1* (**A**), *GSKIP* (**B**), *CBL* (**C**) and *WDR81* (**D**) in adrenal gland in association with genotypes for their respective SNP. Number of samples is shown under each genotype. Risk allele for each genotype is determined by a positive score for GWAS Beta

We hypothesize that these genes might play a role in neuronal differentiation. Specifically, *ZMYM1*, *GSKIP,* and *CBL* may be functionally relevant in the early stages of neural progenitors, with their expression subsequently downregulated as differentiation progresses. Conversely, *WDR81* may be involved in supporting the later stages of neuronal differentiation, as its expression is lower in immature cells but increases in the final differentiated tissues (Fig. 2). By evaluating the expression of these genes in high- and low-risk tumors in a dataset of 498 RNAseq data (GEO ID: GSE62564), we found that *GSKIP* is upregulated in high-risk NB patients, whereas *ZMYM1* and *CBL* are upregulated in low-risk patients. Additionally, *WDR81* was also found to be upregulated in low-risk cases (Supplementary Fig. 1). We then evaluated the eQTL risk genotypes of *ZMYM1*, *GSKIP* and *CBL* which showed a significant association with higher expression levels in the adrenal gland (Fig. 3). We therefore suggest that these SNPs may be potential NB risk factors and may modulate associated gene expression during the later stages of differentiation in the adrenal gland, by contributing to less differentiated phenotypes when upregulated. The eQTL risk genotypes of *WDR81* were associated with lower expression levels in the adrenal gland, indicating a possible opposite role in driving dysregulation during the differentiation process (Fig. 3).

Collectively, these findings propose that altered expression of NB risk genes may contribute to impaired differentiation, a primary aspect of NB etiology.

### Single-cell data showed differential expression of identified risk genes in undifferentiated and differentiated neuronal cell types

**Fig. 4:**
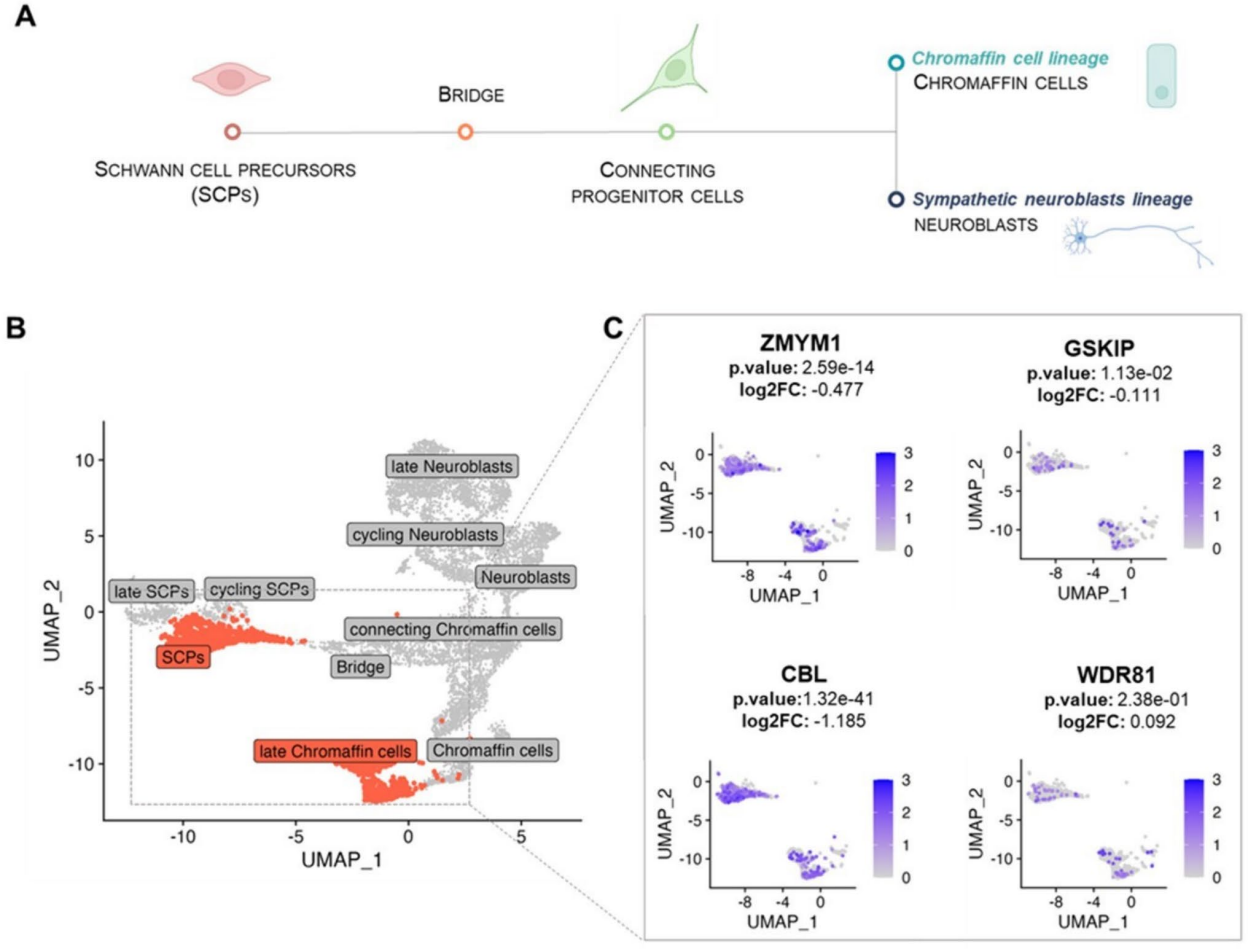
Expression of candidate NB risk genes in undifferentiated and differentiated cell subtypes from fetal adrenal medulla. **A** Schematic representation of lineage trajectories and cell populations during the normal adrenal medulla development. **B** UMAP (Uniform Manifold Approximation and Projection) plot showing cell populations normally found during adrenal medulla development. Cell populations representing the initial and final stages of differentiation selected for our investigation are highlighted in red. **C** Zoom in of the UMAP plots showing the gene expression of the candidate genes in the two selected populations. Dots (cells) are filled according to the color scale indicated on each panel. P-values and log2FC comparing late Chromaffin cells versus SCPs are also reported. SCPs, Schwann-cell precursors; p.value, false discovery rate (FDR); log2FC, Fold Change

We exploited single-cell transcriptomic data from normal developing human adrenal glands sourced from public data (Jansky et al. 2021) and investigated candidate genes expression in cell population trajectories during normal adrenal gland development (Fig. 4A).

We focused on the initial and final stages of cellular differentiation, comparing SCPs, representative of early stages, with chromaffin cells, indicative of the late stages (Fig. 4B). Our analysis confirmed that the expression levels of *ZMYM1*, *CBL*, and *GSKIP* decreased as differentiation advances within the adrenal gland (log2FC = – 0.477, log2FC = – 1.185 and log2FC = – 0.111, respectively), even if only *ZMYM1* and *CBL* reached statistical significance after multiple testing correction (FDR = 2.59e-1 and 1.32e-41). Notably, the most undifferentiated cell type, reminiscent of neuronal precursor cells (SCPs) showed higher expression levels of these genes compared to cell types proceeding towards the final stages of differentiation, such as late chromaffin cells (Fig. 4C). In contrast, *WDR81* did not exhibit significant expression differences (Fig. 4C). To address additional evidences showing that some NBs may resemble neuroblasts and may also originate from neuroblast lineage (Korber et al. 2023), we performed the same analysis comparing candidate genes’ expression levels between SCPs and late neuroblasts. Of note, *ZMYM1* and *CBL* were significantly downregulated in the differentiated subtypes (Supplementary Fig. 2), in line with what we observed in the previous analysis; *GSKIP* and *WDR81* were not significantly modulated (Supplementary Fig. 2).

We speculate that the different expression patterns of these genes may be linked to specific differentiation stages. Consequently, their dysregulation, potentially influenced by genetic variants associated with NB risk, could contribute to impaired differentiation and have a decisive role in NB development.

### In silico analysis of gene expression in a differentiated NB cell line induced by RA

To strengthen the hypothesis of the potential involvement of the identified risk genes in differentiation processes, we conducted in silico analysis of the expression levels of *ZMYM1*, *GSKIP*, *CBL*, and *WDR81* from NB cells treated with RA. The linear regression analysis performed on genes’ expression data from RA-induced differentiation experiment in the NB cell line SH-SY5Y revealed distinct trends. *ZMYM1* showed a downregulation from day 1 to 5 of RA treatment (P-value: 0.002) (Supplementary Fig. 3). *GSKIP* exhibited a downregulation only at day 2, while its expression increases at later time-points, displaying a regulation in its expression that is in contrast with our initial hypothesis; however, this pattern of expression did not reach significance level in linear regression analysis (P-value: 0.14) (Supplementary Fig. 3). Similarly, *CBL* showed a decrease in expression with a more pronounced decline at day 3 (P-value: 0.14) (Supplementary Fig. 3). Conversely, *WDR81* levels significantly increase up to 5 days of RA treatment (P-value: 0.0003) (Supplementary Fig. 3). Overall, consistent with our initial hypothesis, both *ZMYM1* and *CBL* showed downregulation as the differentiation treatment progressed, suggesting a reduced requirement for their expression in the later stages of differentiation. In contrast, *WDR81* exhibited an opposite modulation, with increasing expression levels as the differentiation progressed. Conversely, *GSKIP* expression pattern was not consistent with our initial hypothesis.

### In vitro RA-induced differentiation in NB cell lines resulted in *ZMYM1*, *GSKIP* and *CBL* downregulation, while *WDR81* showed upregulated expression

In order to confirm our in silico predictions, we performed differentiating experiments using RA in two NB cell lines, SH-SY5Y and SK-N-BE(2). We selected these cell lines since they exhibited basal expression of the identified NB risk genes (Supplementary Fig. 4) and are already known to be responsive to RA induction (Thiele). Notably, we observed very low levels of both WDR81 protein and mRNA in all tested NB cell lines (Supplementary Fig. 4). Nevertheless, we proceeded with further evaluation on this gene since our data predicted a potential increase in its expression during the differentiation process.

The RA-induced differentiation outcome was confirmed by observing changes in cell morphology, determined by the extension of multiple cellular processes in RA-treated cells (Supplementary Fig. 5A), and an increase in the expression of neuronal markers (i.e. NEFL, NEUN, Synaptophysin, Tau) in both cell lines (Supplementary Fig. 5B).

**Fig. 5:**
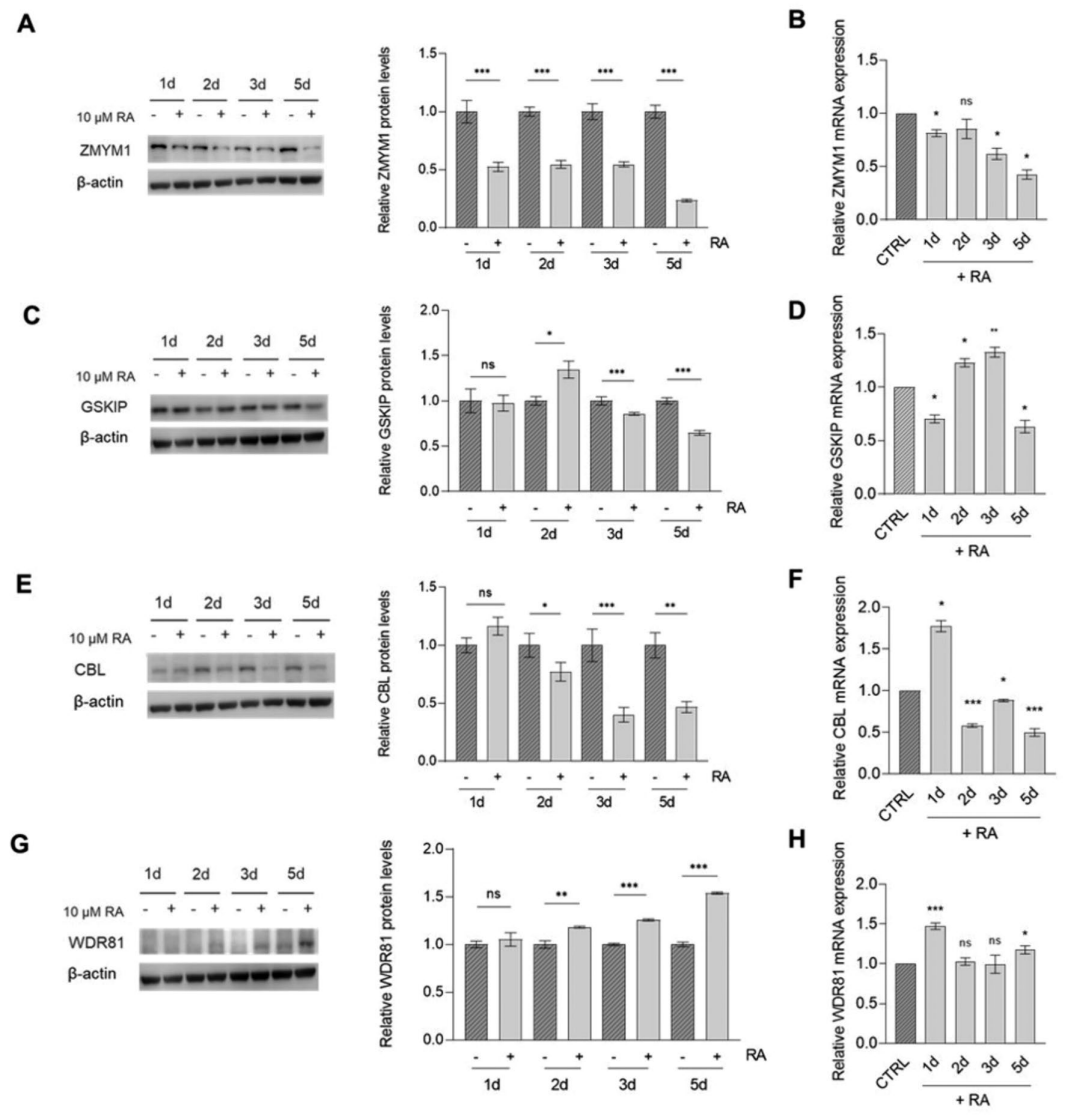
Candidate NB risk genes expression in SH-SY5Y after RA-induced differentiation. Protein levels of *ZMYM1* (**A**), *GSKIP* (**C**), *CBL* (**E**) and *WDR81* (**G**) were determined through western blot (left) and evaluated through densitometry (right) in SH-SY5Y cell line following treatment with 10 µM RA for 5 days. β-actin was used as loading control. mRNA levels of *ZMYM1* (**B**), *GSKIP* (**D**), *CBL* (**F**) and *WDR81* (**H**) in SH-SY5Y cell line following treatment with 10 µM RA for 5 days were determined through qPCR and represented as fold-change on control (represented as CTRL bar). Data are shown as mean ± standard deviation of technical duplicates from three independent experiments. Datapoints: 1d (1 day), 2d (2 day), 3d (3 day) and 5d (5 day). P-value by Student t-test, ***p < 0.001, **p < 0.01, *p < 0.05. CTRL: control; ns: not significant

Subsequently, we investigated the expression of candidate genes in response to RA treatment, to assess their potential involvement in pathways associated with neuronal differentiation. In SH-SY5Y, *ZMYM1* and *CBL* exhibited a significant downregulation in both protein and mRNA levels (Fig. 5A, B and E, F).

In contrast, *GSKIP* showed a different modulation, with decrease in expression observed only at later time point (day 5) (Fig. 5C, D). *WDR81* exhibited increased expression, primarily at the protein level (Fig. 5G, H).

**Fig. 6:**
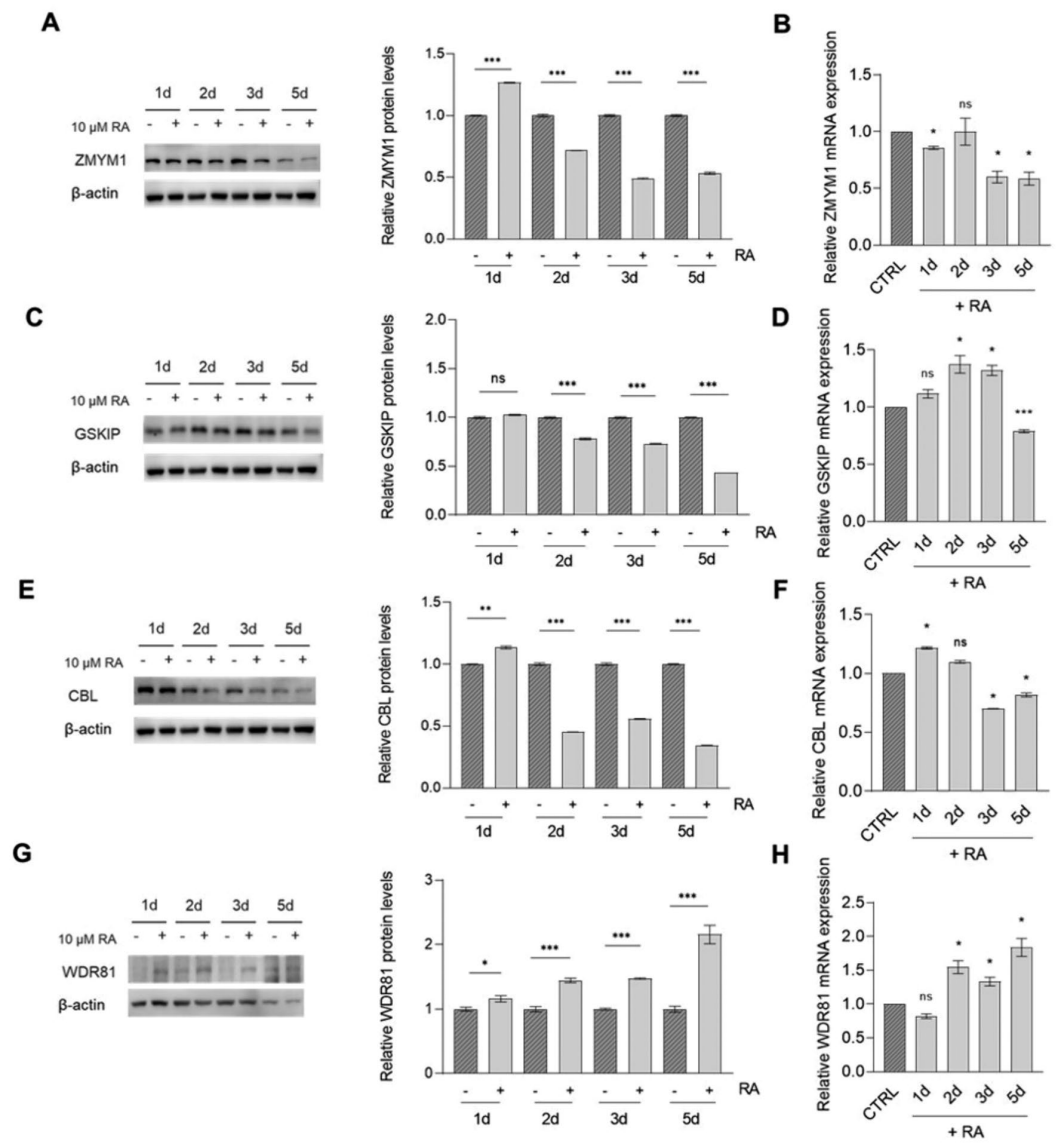
Candidate NB risk genes expression in SK-N-BE(2) after RA-induced differentiation. Protein levels of *ZMYM1* (**A**), *GSKIP* (**C**), *CBL* (**E**) and *WDR81* (**G**) were determined through western blot (left) and evaluated through densitometry (right) in SK-N-BE(2) cell line following treatment with 10 µM RA for 5 days. β-actin was used as loading control. mRNA levels of *ZMYM1* (**B**), *GSKIP* (**D**), *CBL* (**F**) and *WDR81* (**H**) in SK-N-BE(2) cell line following treatment with 10 µM RA for 5 days were determined through qPCR and represented as fold-change on control (represented as CTRL bar). Data are shown as mean ± standard deviation of technical duplicates from three independent experiments. Datapoints: 1d (1 day), 2d (2 day), 3d (3 day) and 5d (5 day). P-value by Student t-test, ***p < 0.001, **p < 0.01, *p < 0.05. *CTRL* control., *ns* not significant

Subsequently, we performed RA-induced differentiation in SK-N-BE(2) cells. Concordantly to SH-SY5Y, *ZMYM1* and *CBL* exhibited a significant decrease in both protein and mRNA levels during differentiation **(**Fig. 6 A, B and E, F)

*GSKIP* and *WDR81* displayed comparable patterns of regulation to those observed in SH-SY5Y. Indeed, *GSKIP* demonstrated a clear downregulation at day 5 of differentiation (Fig. 6C, D) while *WDR81* levels increased during the differentiation process (Fig. 6G, H).

Taken together, these findings suggest that these genes may be differentially modulated during RA treatment and might be implicated in pathways converging to neuronal differentiation. Consequently, the deregulation of their expression levels in different stages of neuronal differentiation could potentially impact the initiation of NB*.*

### The differentiation treatment with BDNF further supported the modulation of candidate NB risk genes

To validate our in vitro findings, we employed an alternative differentiating protocol. We cultured NB cell lines with BDNF for 3 days after RA withdrawal on day 5 of RA treatment, inducing the transition to a more differentiated, neuron-like phenotype (Encinas et al. 2000; Hromadkova et al. 2020). Both SH-SY5Y and SK-N-BE(2) cells treated with BDNF displayed distinctive neuronal morphology when compared to control cells, characterized by the extension of multiple neurite-like cellular processes and synaptic branching (Supplementary Fig. 6A). The gain of a fully differentiated phenotype was further verified by the assessment of neuronal markers (Supplementary Fig. 6B–E).

**Fig. 7:**
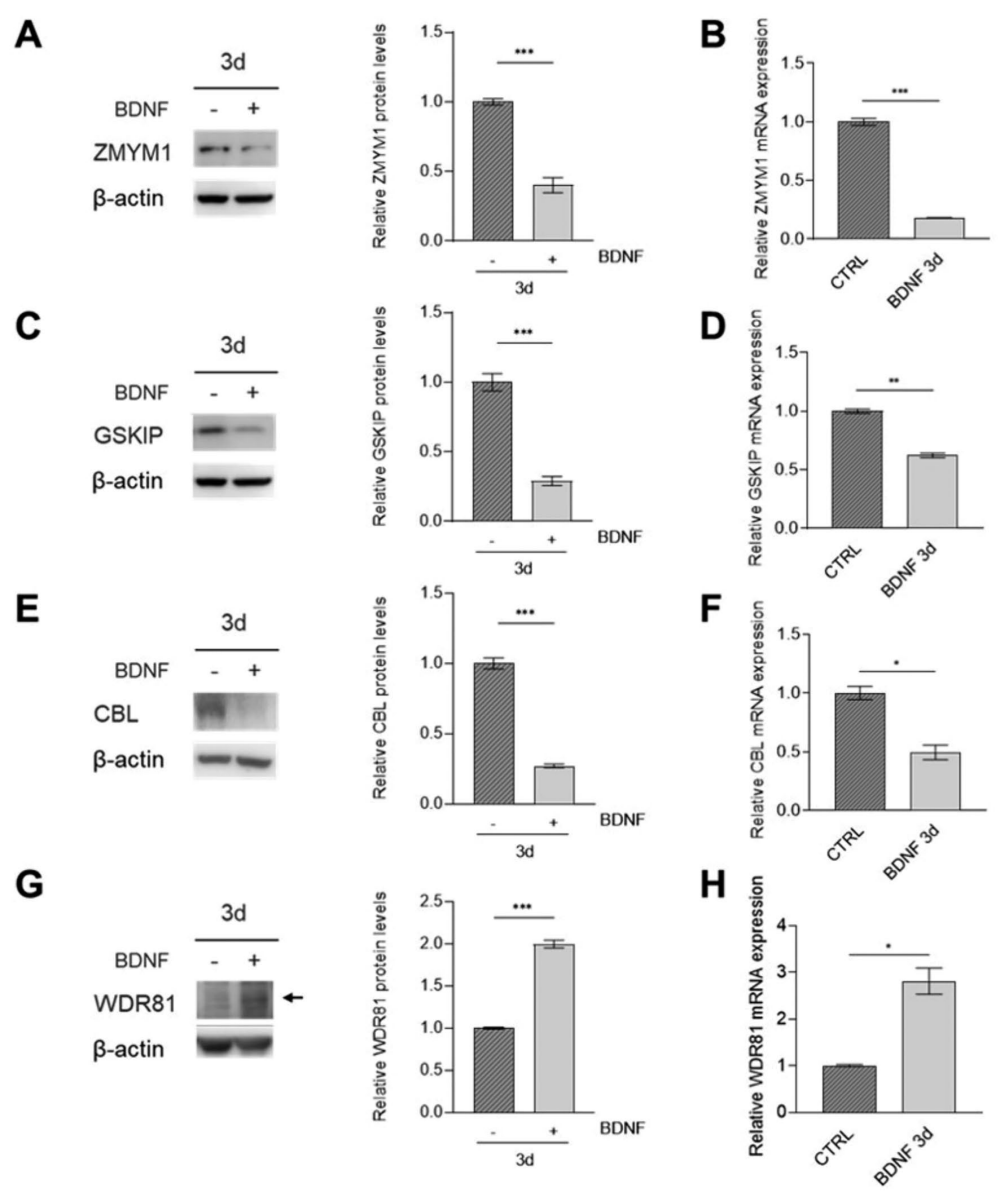
Candidate NB risk genes expression in SH-SY5Y after BDNF-induced differentiation. Protein levels of *ZMYM1* (**A**), *GSKIP* (**C**), *CBL* (**E**) and *WDR81* (**G**) were determined through western blot (left) and evaluated through densitometry (right) in SH-SY5Y cell line following addition of BDNF for 3 days, after treatment with 10 µM RA for 5 days. β-actin was used as loading control. mRNA levels of *ZMYM1* (**B**), *GSKIP* (**D**), *CBL* (**F**) and *WDR81* (**H**) in SH-SY5Y cell line following addition of BDNF for 3 days, after treatment with 10 µM RA for 5 days were determined through qPCR and represented as fold-change on control (represented as CTRL bar). Data are shown as mean ± standard deviation of technical duplicates from three independent experiments. Datapoint: 3d (3 day). P-value by Student t-test, ***p < 0.001, **p < 0.01, *p < 0.05. CTRL: control

In SH-SY5Y cells, protein and mRNA levels evaluation revealed a significant decrease in the expression of *ZMYM1*, *CBL*, and *GSKIP* following BDNF treatment (Fig. 7A–F).

In contrast, *WDR81* exhibited an upregulation in response to BDNF treatment (Fig. 7G, H).

**Fig. 8:**
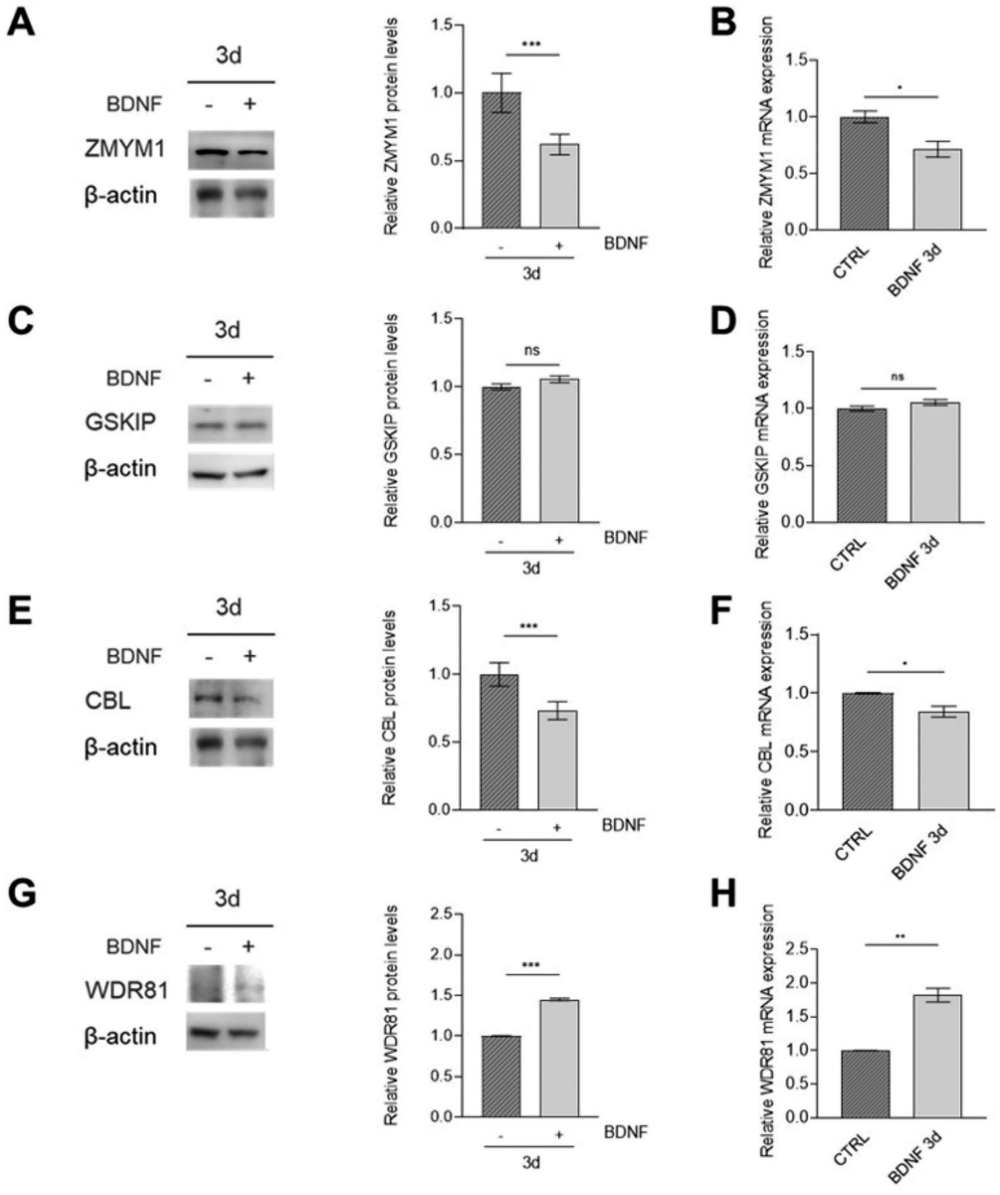
Candidate NB risk genes expression in SK-N-BE(2) after BDNF-induced differentiation. Protein levels of *ZMYM1* (**A**), *GSKIP* (**C**), *CBL* (**E**) and *WDR81* (**G**) were determined through western blot (left) and evaluated through densitometry (right) in SK-N-BE(2) cell line following addition of BDNF for 3 days, after treatment with 10 µM RA for 5 days. β-actin was used as loading control. mRNA levels of *ZMYM1* (**B**), *GSKIP* (**D**), *CBL* (**F**) and *WDR81* (**H**) in SK-N-BE(2) cell line following addition of BDNF for 3 days, after treatment with 10 µM RA for 5 days were determined through qPCR and represented as fold-change on control (represented as CTRL bar). Data are shown as mean ± standard deviation of technical duplicates from three independent experiments. Datapoint: 3d (3 day). *p*-value by Student t-test, ***p < 0.001, **p < 0.01, *p < 0.05, ns: not significant. CTRL: control

In SK-N-BE(2) cells, we observed less pronounced changes in genes expression. Indeed, modest reduction in the expression of *ZMYM1* and *CBL* at both protein and mRNA levels was distinguishable (Fig. 8A, B and E, F), while *GSKIP* levels did not show significant differences between treated and control cells (Fig. 8C, D).

However, similarly to SH-SY5Y, *WDR81* displayed increased expression levels with BDNF treatment in SK-N-BE(2) (Fig. 8G, H).

These results further validated that the expression of the identified NB risk genes might be modulated during differentiating processes, although the extent of these changes varies between different cell lines and under different treatments.

## Discussion

GWASs have become invaluable tools in the investigation of complex diseases, aiding in the identification of genetic variants potentially linked to disease initiation and progression. However, most of them are found in non-coding regions of the genome, which primarily have regulatory functions. Consequently, relying on GWASs findings alone often fails to precisely identify the genes responsible for a condition and to offer a comprehensive understanding of the functional and biological significance of the identified variants. To overcome this limitation, the integration of different data types has emerged as a promising approach to gain deeper insights into the genetic mechanisms underlying complex traits.

In this context, we presented the findings from our TWAS approach applied to NB. We employed a colocalization analysis using a Bayesian method implemented in the Sherlock tool (He et al. 2013). This approach was based on the integration of GWAS data from a cohort of NB patients and eQTL data from the adrenal gland, which represents the tissue where NB preferentially arises. As a result, our study showed seven SNPs associated with NB susceptibility, also linked with the dysregulation of specific target genes. To strengthen the reliability of our findings, we further validated these results using an alternative method, a TWAS with MetaXcan employing MASHR prediction models (Barbeira et al. 2017). This additional evaluation further pinpointed the potential involvement of these SNPs in the regulation of their respective target genes in the context of NB.

To deepen the knowledge about candidate genes’ contribution in NB, we examined their expression levels in silico, using data from NB tumors, neural crest cells, neural precursors cells, differentiated tissues, and single-cell data from normal adrenal gland development. We observed that four of these genes, namely *ZMYM1*, *CBL*, *GSKIP*, and *WDR81*, exhibited dysregulation in NB tumors. Notably, *ZMYM1*, *CBL*, and *GSKIP* exhibited lower expression levels in differentiated tissues, compared to neural crest and neural precursors cell lines and tumoral tissues, while *WDR81* showed opposite trend of expression. Accordingly, risk alleles for *ZMYM1*, *CBL* and *GSKIP* correlated with higher expression in adrenal gland, while risk allele for *WDR81* correlated with lower expression in the same tissue. Single-cell data confirmed that the expression of *ZMYM1*, *CBL*, and *GSKIP* was lower in more differentiated cell types, i.e., late chromaffin cells and late neuroblasts, further supporting their potential involvement in the neuronal differentiation, a process essential for NB development. However, in an RNA-seq dataset of NBs, we observed higher expression of *ZMYM1* and *CBL* in low-risk tumors compared to high-risk. This finding was unexpected, as higher-risk NB is typically associated with a less differentiated phenotype, leading us to anticipate that these genes would be upregulated in high-risk NB patients. These differential expression patterns suggest that *ZMYM1* and *CBL* might operate through distinct regulatory mechanisms or interact with other molecular factors in a risk-dependent manner. To elucidate the precise molecular mechanisms by which these genes influence NB pathogenesis and risk stratification, further research is necessary. Future studies should focus on investigating the interactions of these genes with other signaling pathways and their roles in different NB subtypes and stages of differentiation.

Accumulated evidence suggests that halted differentiation of neural crest-derived sympathoadrenal lineage, coupled with oncogenic mutations, may underlie NB formation, influencing its clinical heterogeneity, spontaneous regression and response to differentiation-inducing therapies (Zeineldin et al. 2022). Consequently, any changes or alterations in these genes may potentially contribute to malignant transformation observed in NB.

Each of the identified NB risk genes, *ZMYM1*, *CBL*, *GSKIP*, and *WDR81*, shows functions that make them potential contributors to NB onset. *ZMYM1* is a zinc-finger protein which may act as a transcription factor, with similarities to other MYM-type zinc finger proteins that have been associated with developmental disorders (Graham-Paquin et al. 2023; Hiatt et al. 2023). CBL, specifically c-Cbl, serves as an E3 ubiquitin-protein ligase responsible for promoting the degradation of numerous protein targets, including those associated with receptor tyrosine kinase signaling, such as the epidermal growth factor receptor (EGFR), anaplastic lymphoma kinase (ALK), and TrkA (Thien and Langdon 2001; Motegi et al. 2004; Cooper et al. 2015). Mutations in *Cbl* have been linked to various tumors, particularly myeloid neoplasms, establishing Cbl-encoded protein as oncogene and driver of cancer (Leardini et al. 2022). Specifically, recent studies have demonstrated that reduced levels of Cbl contribute to neurite outgrowth in NB cell lines by sustaining ERK phosphorylation, highlighting its potential in modulating differentiation in NB (Emdal et al. 2015; Pedersen et al. 2021).

GSKIP acts as a negative regulator of GSK3β in the Wnt signaling pathway, influencing vital cellular processes like differentiation, proliferation, and tumor formation (Chou et al. 2006; Duda et al. 2020). Also, GSKIP involvement in regulating neurite outgrowth in SH-SY5Y cells has been observed (Lin et al. 2009). Notably, GSK3β, regulated by GSKIP, has been shown to participate in the specification of axons and dendrites (Luo 2012), and to phosphorylate and stabilize MYCN (Duffy et al. 2014; Izumi et al. 2020). Moreover, GSK3β inhibition has shown potential in reducing neuroblastoma cell viability and inducing cell death (Dickey et al. 2011).

Lastly, *WDR81* mutations have been associated with developmental disorders including cerebellar ataxia, mental retardation, quadrupedal locomotion syndrome (CAMRQ2), and microcephaly (Gulsuner et al. 2011; Alazami et al. 2015). WDR81 is known to regulate endosome-lysosome pathways and endosome trafficking, that is crucial in determining cell fate, migration and neuronal polarity (Wang et al. 2021). Additionally, a recent study has demonstrated that depletion of *WDR81* in adult neural progenitor cells markedly reduced adult hippocampal neurogenesis and impaired hippocampus-dependent learning (Wang et al. 2021).

These findings collectively suggest that the identified genes may be involved in neuronal differentiation and neuronal development, and that their dysregulation due to genetic variants could contribute to NB risk.

Therefore, understanding the role of these genes is central considering that the induction of neuronal differentiation is a therapeutic approach for NB. Retinoic acid derivatives, such as RA, have been used to promote differentiation in cell cultures and are a standard treatment for high-risk NB patients (Matthay et al. 1999; Peinemann et al. 2017). To further explore whether the identified NB risk genes are modulated during RA-induced differentiating treatment, we performed time-course experiments on two NB cell lines, SH-SY5Y and SK-N-BE(2). Our observations revealed *ZMYM1* and *CBL* downregulation in both cell lines. *GSKIP* exhibited reduced expression primarily at later time points, suggesting that higher expression may be necessary during early precursor cell stages, thus decreasing in more mature cell types. Conversely, *WDR81* levels increased during RA treatment.

To demonstrate the sensitivity of these genes to neuronal differentiation process, we performed similar differentiating experiments using the RA-BDNF protocol. BDNF is a neurotrophin playing a crucial role in synaptic connection development by binding to Trk receptors and activating downstream signaling pathways, including PI3K-Akt, which influence axon formation and elongation (Atwal et al. 2000; Menager et al. 2004). While RA treatment alone can result in minimal neurite formation in NB cells, the two-step RA-BDNF protocol more closely mimics neuronal differentiation. As anticipated and consistent with our RA-induced differentiation findings, both *ZMYM* and *CBL* were downregulated after RA-BDNF treatment in both cell lines, with a greater change in SH-SY5Y, while *WDR81* expression increased. Conversely, *GSKIP* exhibited decreased expression only in SH-SY5Y, suggesting that its role may be influenced by the cellular and biological context and could be implicated in pathways regulated by other key effectors. Notably, SK-N-BE(2) cells, unlike SH-SY5Y, harbor *MYCN* amplification, a factor known to be pivotal in NB biology by inhibiting differentiation and maintaining pluripotency (Huang and Weiss 2013). As an example, the activation of GSK3β, a protein regulated by *GSKIP*, is linked to reduced MYCN activation and expression, and its knockdown is sufficient to reduce NB cell viability (Dickey et al. 2011). Hence, we may speculate that the elevated levels of *GSKIP* in SK-N-BE(2) cells treated with BDNF could be due to distinct MYCN-dependent feedback mechanisms for tumor maintenance in a MYCN-amplified context. Additionally, the simultaneous activation of Trk receptors by BDNF might converge on the same final signaling pathways involving PI3K/Akt and GSK3β (Duffy et al. 2014).

Our study presents the first comprehensive analysis that identifies *ZMYM1*, *CBL*, *GSKIP*, and *WDR81* as potential NB risk genes through an integrative genomic approach combining GWAS and eQTL data. While some of these genes have been previously studied in related contexts, we provide novel insights into their collective role in neuronal differentiation within the context of NB pathogenesis. This integrated analysis not only reinforces the potential importance of previously studied genes like *CBL* and *GSKIP* in NB, but also introduces *ZMYM1* and *WDR81* as new candidates in this disease context, thereby expanding our understanding of the genetic landscape and molecular mechanisms involved in NB development. Future experiments are needed to unravel the molecular mechanisms underlying the genetic associations of these genes with NB development and progression.

Although our TWAS, along with additional in silico and in vitro analyses, strongly supports the role of the NB risk genes identified using eQTLs from adult adrenal glands, we acknowledge the necessity for future studies to incorporate eQTL data from fetal or embryonic tissues, when available, to more accurately model the developmental origins of NB.

Additionally, it would be worthy to validate our findings across different populations and ensure broader applicability. This approach will also help to identify population-specific risk variants and improve our understanding of NB pathogenesis on a global scale.

In summary, our study described genes whose dysregulation, influenced by specific SNPs, may play a significant role in NB development. Alterations in the regulation of these genes could impact the differentiation of neural-crest derived precursors in the sympathoadrenal lineage, by determining gene expression changes in specific developmental stages and potentially contributing to NB formation. Importantly, our findings emphasize the value of a comprehensive approach that merges genetic data from GWAS with expression levels and other -*omic* data, to integrate genetic information that often remain poor of biological significance and interpretation. This approach not only sheds light on the intricate mechanisms involved in complex diseases like NB, but also identifies previously unknown genes implicated in the disease pathophysiology which could represent novel potential therapeutic targets.

## Supplementary Information

Below is the link to the electronic supplementary material.

Supplementary file1 (DOCX 2988 KB)

## Data Availability

No datasets were generated or analysed during the current study.
