# Supplementary material for: Integrative genomic analyses identify neuroblastoma risk genes involved in neuronal differentiation
Source: Hum Genet. 2024 Aug 27;143(11):1293–309. doi: 10.1007/s00439-024-02700-2 (PMC11522082; doi:10.1007/s00439-024-02700-2)
Supplement: Supplementary file 1 — Supplementary file1 (DOCX 2988 KB) [file 439_2024_2700_MOESM1_ESM.docx]

**Supplementary Information**

**Integrative genomic analyses identify neuroblastoma risk genes involved in neuronal differentiation**

Matilde Tirelli^1,2^, Ferdinando Bonfiglio^1,2^, Sueva Cantalupo^2^, Annalaura Montella^1,2^, Marianna Avitabile^2^, Teresa Maiorino^1,2^, Sharon J. Diskin^3,4^, Achille Iolascon^1,2^, Mario Capasso*^1,2^

^1^University of Naples Federico II, Department of Molecular Medicine and Medical Biotechnology, 80131 Naples, Italy

^2^ CEINGE Biotecnologie Avanzate Franco Salvatore, 80145 Naples, Italy

^3^Division of Oncology and Center for Childhood Cancer Research, Children's Hospital of Philadelphia, 19104 Philadelphia, USA

^4^Department of Pediatrics, Perelman School of Medicine, University of Pennsylvania, 19104 Philadelphia, USA

***Corresponding author:**

Mario Capasso,

University of Naples Federico II

Department of Molecular Medicine and Medical Biotechnology, Naples, Italy

Via Gaetano Salvatore, 486

80145 Naples Italy

Email: mario.capasso@unina.it

Office: +39 081 37 37 889

Lab: +39 081 37 37 736

Fax +39 081 37 37 804

**Supplementary Table 1. Differential expression analysis of candidate genes.**

| **Gene** | **Group 1** | **Group 2** | **n1** | **n2** | **Statistic** | **P** | **Padj** |
| --- | --- | --- | --- | --- | --- | --- | --- |
| *CBL* | undifferentiated tissues | differentiated tissues | 14 | 366 | 4867 | 1.11E-08 | 2.08E-8 |
|  | undifferentiated tissues | tumor | 14 | 219 | 2152 | 1.20E-02 | 1.38E-2 |
|  | differentiated tissues | tumor | 366 | 219 | 14428 | 1.96E-38 | 1.47E-37 |
| *GSKIP* | undifferentiated tissues | differentiated tissues | 14 | 366 | 4996 | 1.61E-9 | 3.45E-09 |
|  | undifferentiated tissues | tumor | 14 | 219 | 2531 | 4.51E-5 | 6.15E-05 |
|  | differentiated tissues | tumor | 366 | 219 | 16397 | 5.17E-33 | 1.94E-32 |
| *WDR81* | undifferentiated tissues | differentiated tissues | 14 | 366 | 750 | 7.12E-6 | 1.07E-05 |
|  | undifferentiated tissues | tumor | 14 | 219 | 1035 | 4.2E-2 | 4.50E-02 |
|  | differentiated tissues | tumor | 366 | 219 | 55624 | 3.90E-15 | 1.17E-14 |
| *ZMYM1* | undifferentiated tissues | differentiated tissues | 14 | 366 | 5067 | 5.32E-10 | 1.33E-09 |
|  | undifferentiated tissues | tumor | 14 | 219 | 2751 | 6.38E-7 | 1.06E-06 |
|  | differentiated tissues | tumor | 366 | 219 | 11776 | 2.05E-46 | 3.08E-45 |
| *SH3BGR* | undifferentiated tissues | differentiated tissues | 14 | 365 | 1978 | 1.51E-1 | 1.51E-01 |
|  | undifferentiated tissues | tumor | 14 | 219 | 2226 | 5E-3 | 6.25E-03 |
|  | differentiated tissues | tumor | 365 | 219 | 64237 | 9.74E-35 | 4.87E-34 |

Notes: Data were obtained from the R2: Genomics Analysis and Visualization Platform. Undifferentiated tissues: Normal neural crest and Neural precursor cells datasets; Differentiated tissues: Normal adrenal gland and Normal tissues datasets; Tumor: Tumor neuroblastoma datasets and Xenograft neuroblastoma dataset. n1: number of samples of Group 1; n2; number of samples of Group2; Statistic: Wilcoxon-Mann-Whitney test statistic; P=P-value; Padj: FDR adjusted P-value.


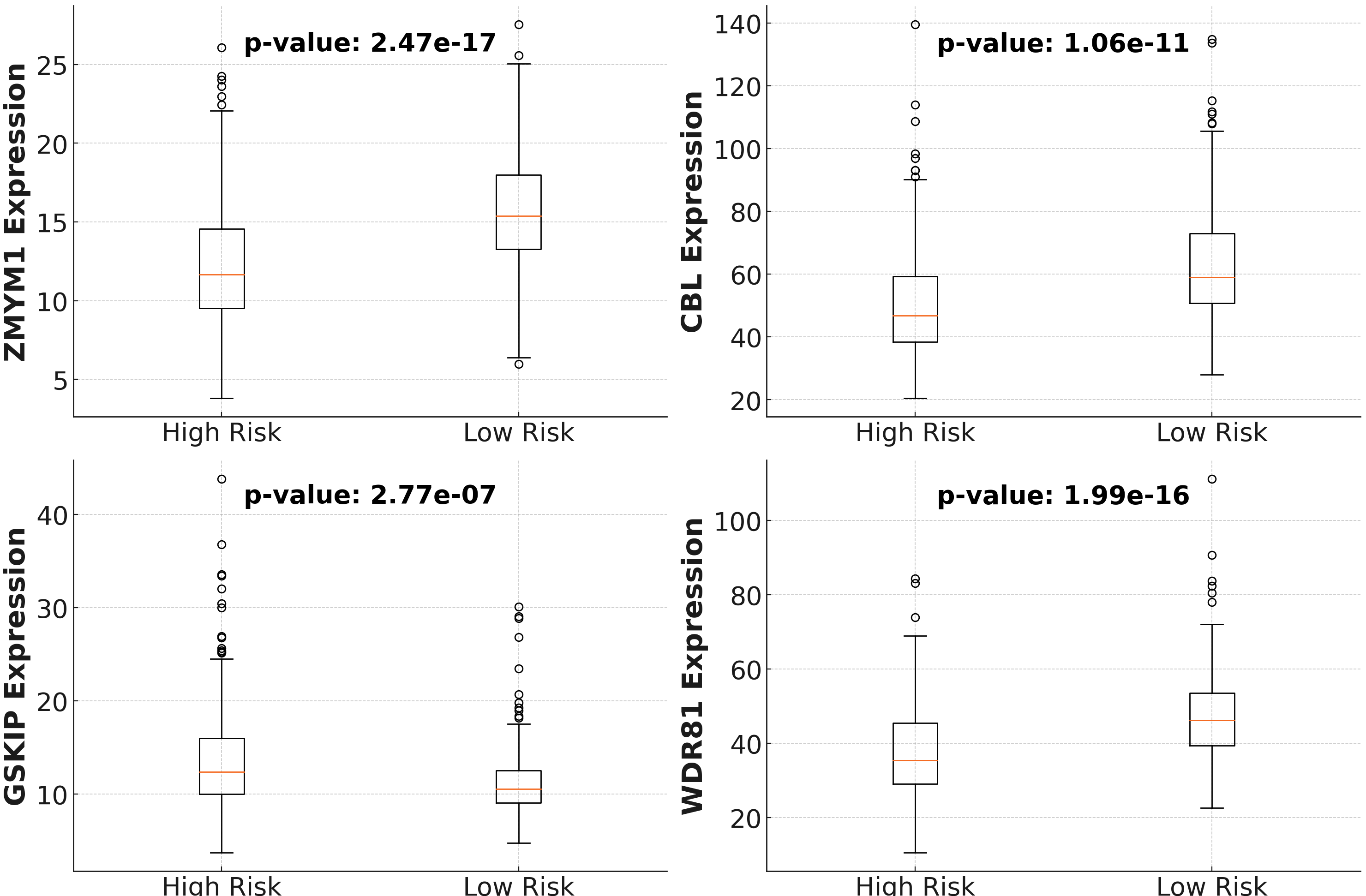


**Supplementary Figure 1.** **Gene expression levels in NB High-Risk and Low-Risk groups.** Boxplots show the expression levels of four genes (*ZMYM1*, *CBL*, *GSKIP*, *WDR81*) between high-risk and low-risk NB groups. Normalized gene expression data of 496 samples, profiled by RNAseq, have been downloaded by R2: Genomics Analysis and Visualization Platform (GEO ID: GSE62564). Statistically significant differences in expression levels between the groups are indicated with p-values from the Mann-Whitney U test.


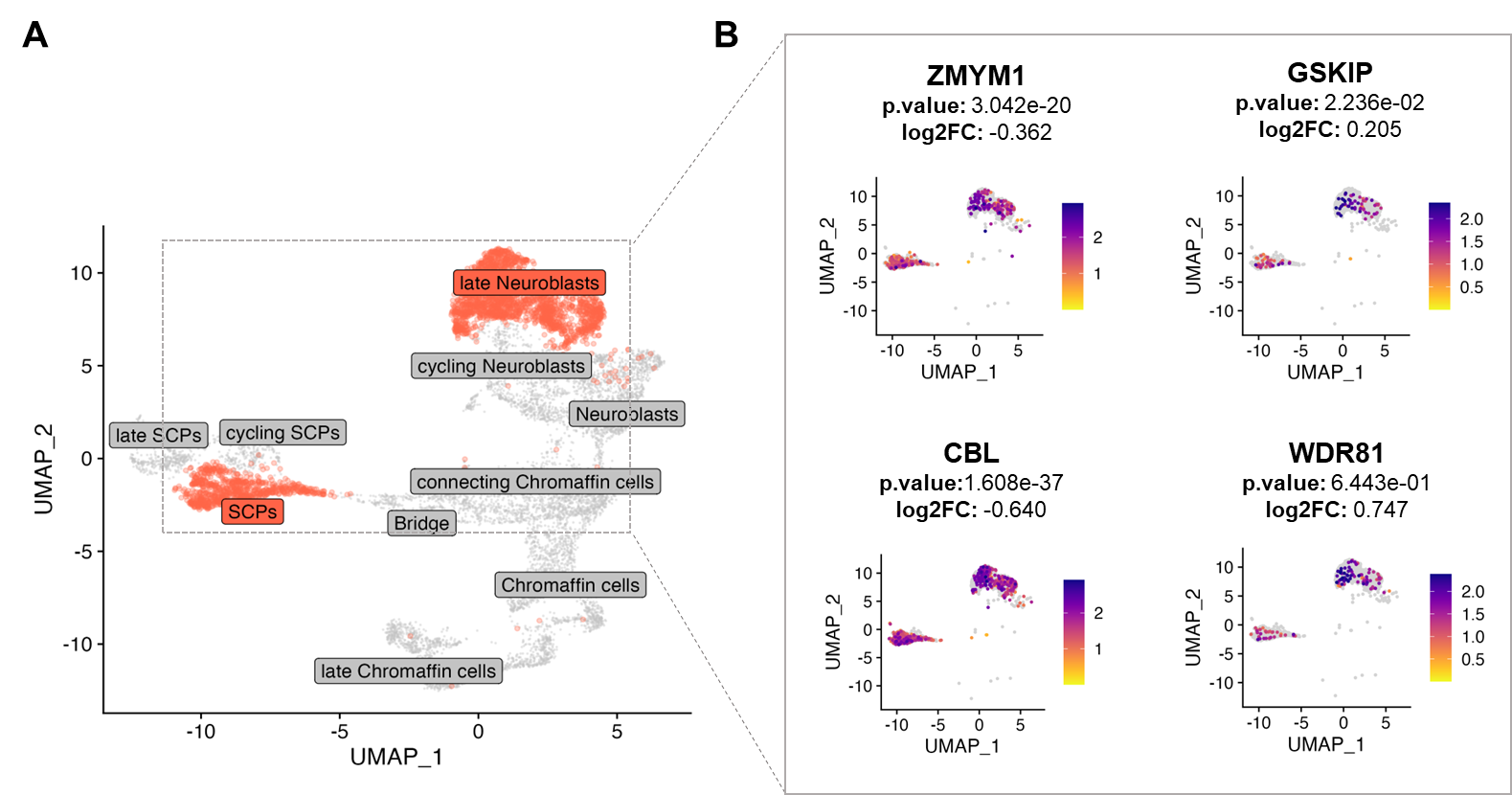


**Supplementary Figure 2. Expression of candidate NB risk genes in undifferentiated (SCPs) and differentiated cell subtypes (late neuroblasts) from fetal adrenal medulla A.** UMAP (Uniform Manifold Approximation and Projection) plot showing cell populations normally found during adrenal medulla development. Cell populations representing the initial and final stages of differentiation selected for our investigation are highlighted in red. **B.** Zoom in of the UMAP plots showing the gene expression of the candidate genes in the two selected populations. Dots (cells) are filled according to the color scale indicated on each panel. P-values and log2FC comparing late Neuroblasts versus SCPs are also reported. SCPs: Schwann-cell precursors. p.value: false discovery rate (FDR); log2FC: Fold Change.

**
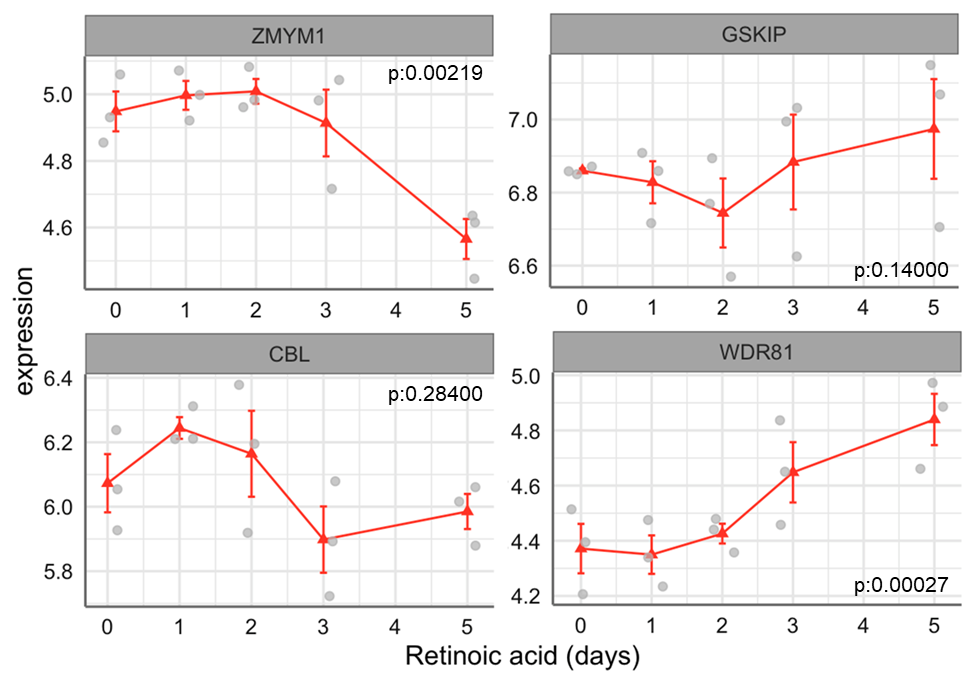
**

**Supplementary Figure 3. Expression of candidate NB risk genes in RA-induced differentiated SH-SY5Y from *in silico* data.** Expression of candidate genes are reported stratified by RA treatment time-points (X axis: time 0, 1 day, 2 days, 3 days, 5 days). Differences between gene expression and treatment time-points were tested using linear regression**.** P=P-value.


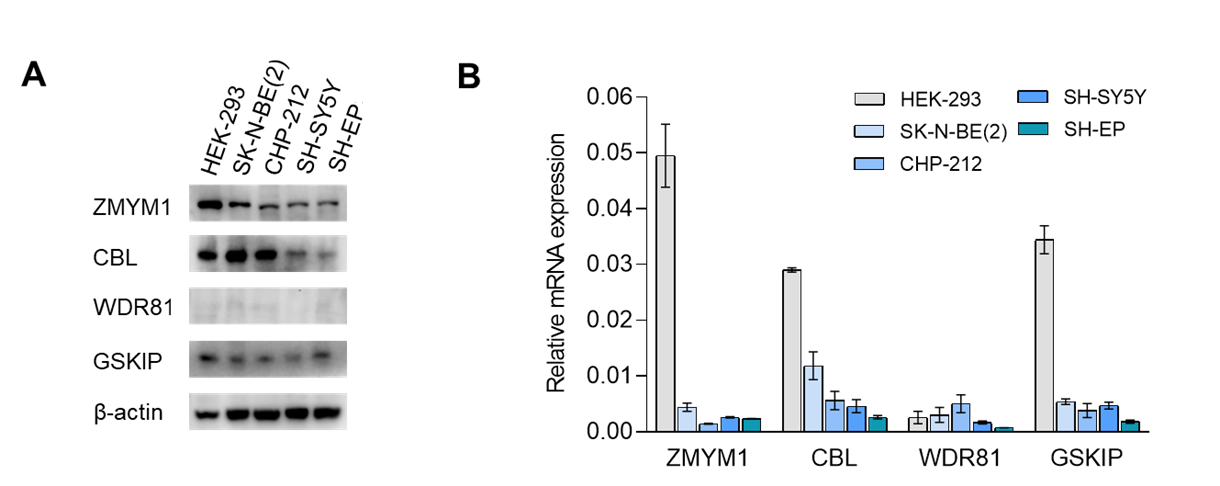


**Supplementary Figure 4. Basal expression levels of candidate NB risk genes. A.** Protein expression levels of ZMYM1, CBL, WDR81, GSKIP in HEK-293 (used as positive control) and NB cell lines (SK-N-BE(2), CHP-212, SH-SY5Y, SH-EP). β-actin is used as loading control. **B.** mRNA expression levels of ZMYM1, CBL, WDR81, GSKIP in HEK-293 (used as positive control) and NB cell lines (SK-N-BE(2), CHP-212, SH-SY5Y, SH-EP). Data are shown as fold-change on β-actin. Data shown are the mean ± standard deviation of technical duplicates from three independent experiments.


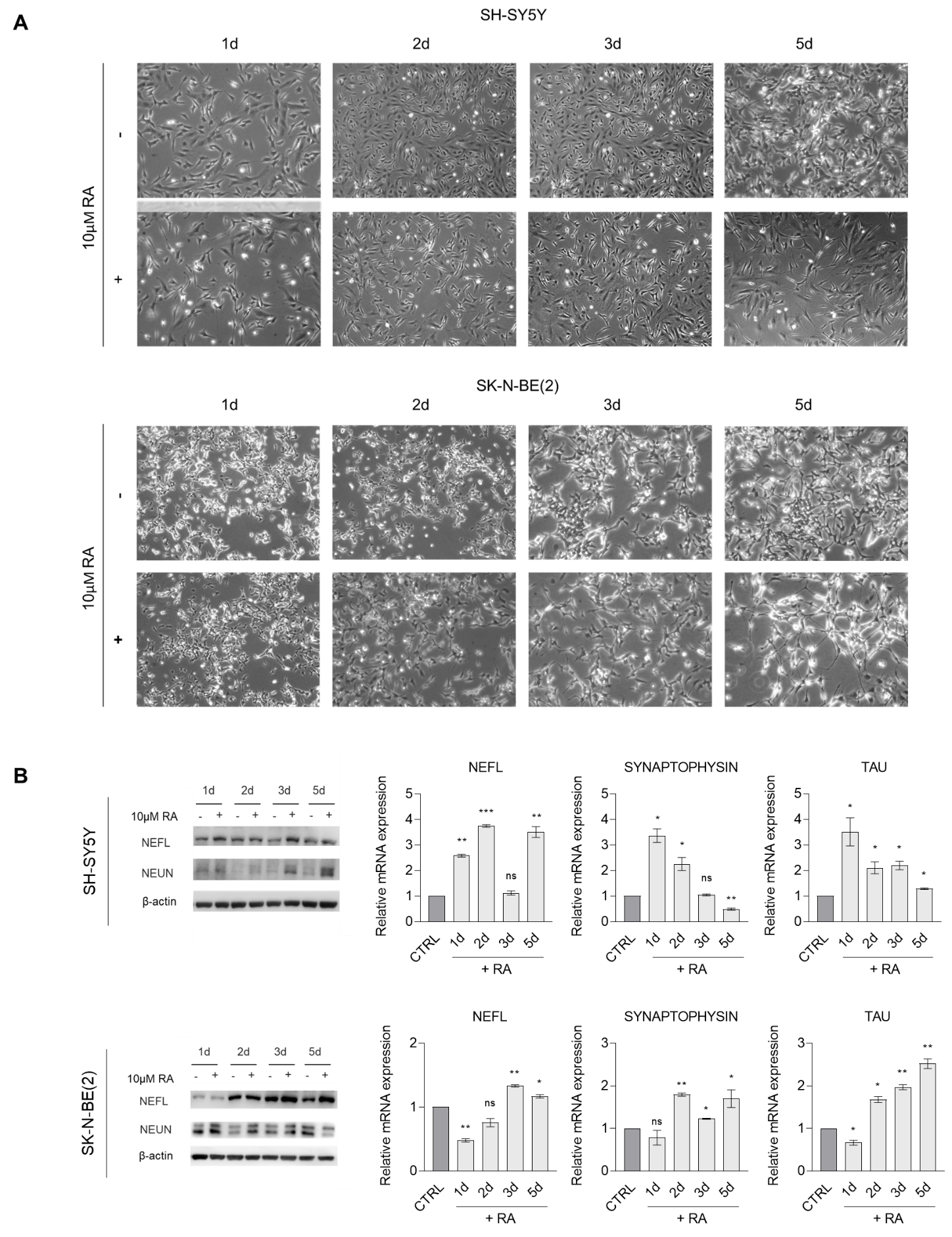


**Supplementary Figure 5. Neuronal differentiation induced by RA in SH-SY5Y and SK-N-BE(2). A.** Representative phase contrast microscopy images showing morphological features of differentiated SH-SY5Y (top) and SK-N-BE(2) (bottom) following treatment with 10 µM RA for 5 days. Neuronal differentiation was assessed through observation of lengthened neurites compared with control (FBS-deprived cells without RA addition). Magnification 10X. Datapoints: 1d (1 day), 2d (2 day), 3d (3day) and 5d (5 day). **B.** Expression of neuronal markers was analysed by western blotting (left) and qPCR (right) in SH-SY5Y (top) and SK-N-BE(2) (bottom) following treatment with 10 µM RA for 5 days. β-actin was used as loading control. qPCR data are shown as mean ± standard deviation of technical duplicates from three independent experiments. The relative mRNA expression is represented as fold-change on control (represented as CTRL bar). Datapoints: 1d (1 day), 2d (2 day), 3d (3day) and 5d (5 day). *p*-value by Student t-test, ***p < 0.001, **p < 0.01, *p < 0.05, ns: not significant. CTRL: control.


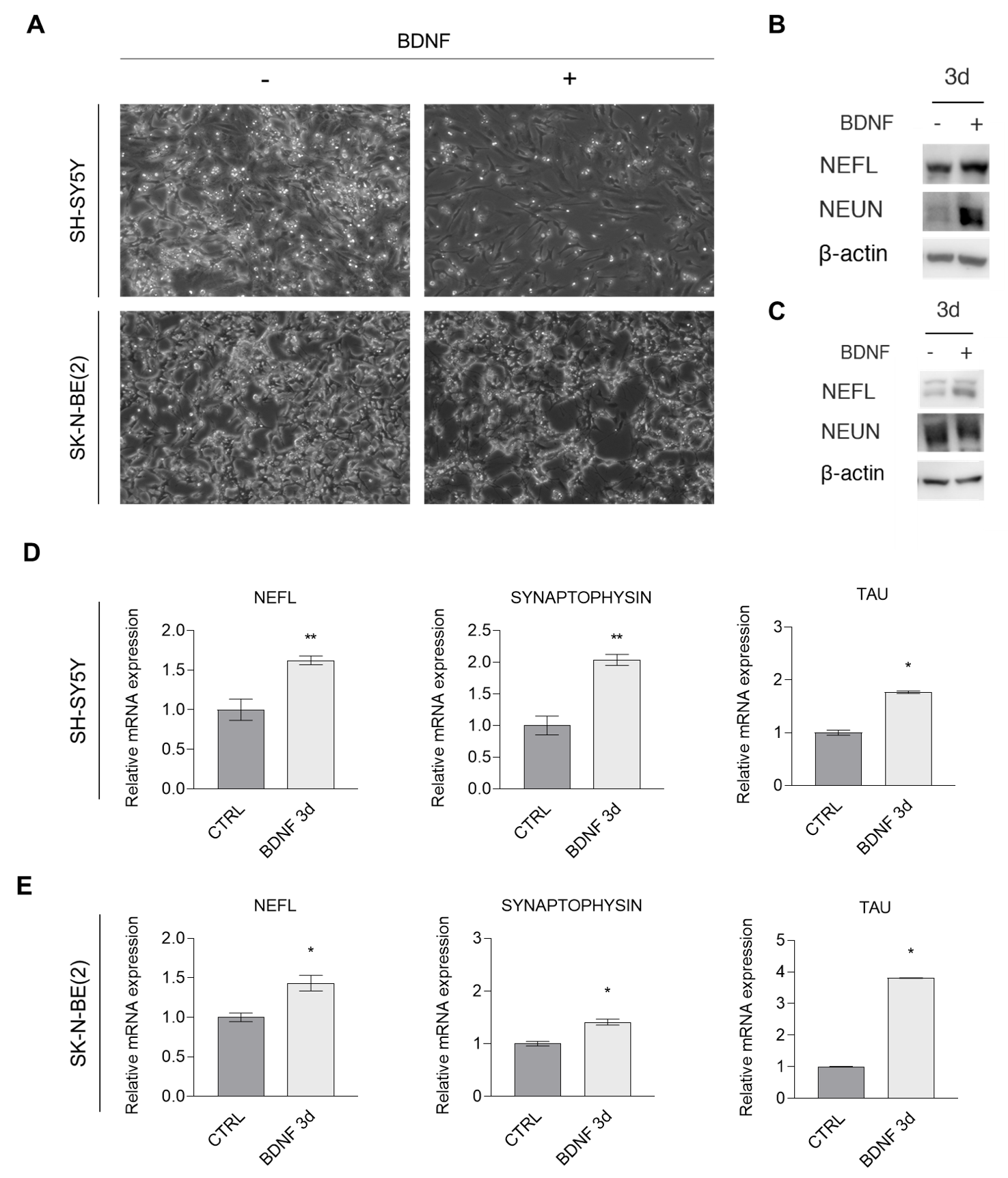


**Supplementary Figure 6. Neuronal differentiation induced by BDNF in SH-SY5Y and SK-N-BE(2). A.** Representative phase contrast microscopy images showing morphological features of differentiated SH-SY5Y (top) and SK-N-BE(2) (bottom) following BDNF addition for 3 days, after treatment with 10 µM RA for 5 days. Neuronal differentiation was assessed through observation of lengthened neurites compared with control (represented as CTRL bar). Magnification 10X. Datapoint: 3d (3day). **B-C.** Expression of neuronal markers was analysed by western blotting (NEFL, NEUN) in SH-SY5Y (**B**) and SK-N-BE(2) (**C**) following BDNF treatment compared to control cells. β-actin was used as loading control. **D-E.** qPCR of neuronal markers in SH-SY5Y (**D**) and SK-N-BE(2) (**E**) following BDNF-induced differentiation. qPCR data are shown as mean ± standard deviation of technical duplicates from three independent experiments. The relative mRNA expression is represented as fold-change on control (represented as CTRL bar). Datapoints: 3d (3day). P-value by Student t-test, **p < 0.01, *p < 0.05. CTRL: control.
